# Long-term use of lurasidone in patients with bipolar disorder: safety and effectiveness over 2 years of treatment

**DOI:** 10.1186/s40345-017-0075-7

**Published:** 2017-03-02

**Authors:** Andrei Pikalov, Joyce Tsai, Yongcai Mao, Robert Silva, Josephine Cucchiaro, Antony Loebel

**Affiliations:** Sunovion Pharmaceuticals Inc., One Bridge Plaza North, Suite 510, Fort Lee, NJ 07024 USA

**Keywords:** Bipolar disorder, Major depressive episode, Atypical antipsychotic, Maintenance treatment, Lurasidone

## Abstract

**Background:**

Bipolar disorder is a chronic illness with a 2-year recurrence rate of approximately 50% among individuals receiving treatment in the community. The aim of this 18-month, open-label, continuation study was to evaluate the long-term safety and effectiveness of lurasidone in patients who initially presented with a major depressive episode associated with bipolar disorder, and who had completed at least 6 months of initial treatment with lurasidone.

**Methods:**

Patients with bipolar I depression were enrolled in one of three 6-week, double-blind, placebo-controlled trials (monotherapy with lurasidone, 1 study; adjunctive therapy with lurasidone; and lithium or valproate, 2 studies). Study completers were eligible for a 6-month, open-label extension study of lurasidone utilizing flexible daily doses of 20–120 mg; extension completers were then eligible for an additional 18 months of continuation treatment with flexible, once-daily doses of lurasidone in the range of 20–80 mg. Concomitant therapy with mood stabilizers was permitted throughout the open-label extension and continuation studies.

**Results:**

A total of 1199 patients entered, and 941 (78.5%) completed initial, double-blind, acute treatment, of whom 817/941 (86.8%) entered, and 559 (68.4%) completed the 6-month extension study; 122/559 patients (21.8%) entered the 18-month continuation study, of whom 19.7% of discontinued, including 6.6% due to adverse events and 1.6% due to insufficient efficacy. The mean dose of lurasidone during the 18-month continuation study was 61.8 mg/day, and the modal dose was 60 mg/day. Mean change in weight, from acute baseline to 18-month continuation endpoint was +0.8 kg (completers, *n* = 55); median changes in cholesterol and triglycerides were −3.0 mg/dL and +26.0 mg/dL, respectively. Based on a Kaplan–Meier analysis, the probability of relapse during 18 months of continuation treatment with lurasidone was estimated to be 18.3% in the monotherapy group and 29.1% in the adjunctive therapy group. Improvement in global illness severity was also maintained during 18 months of continuation therapy (CGI-S at continuation baseline, 2.1; 18-month completers, 1.7; LOCF-endpoint, 1.9).

**Conclusions:**

Up to 2 years of treatment with lurasidone was safe and well tolerated in this bipolar disorder population presenting with an index episode of depression. Improvement in depressive symptoms was maintained in the majority of patients treated with lurasidone, with relatively low rates of relapse, and with minimal effects on weight and metabolic parameters.

## Background

Among patients with a diagnosis of bipolar I disorder, episodes typically recur over many decades (Suppes et al. 2000; Marneros and Brieger 2002; Angst et al. 2003) with episodes of depression occurring with greater frequency than episodes of mania (Judd et al. 2002; Kupka et al. 2007). The persistent risk of recurrent depression and the marked degree of depression-related disability have led to guideline recommendations that emphasize the importance of maintenance therapy (Grunze et al. 2013; Yatham et al. 2013; NICE 2014).

Current guidelines for the maintenance treatment of bipolar disorder recommend lithium, valproate, olanzapine, and quetiapine as first-line monotherapies, and combination therapy with quetiapine and lithium or valproate as first-line adjunctive therapies (Yatham et al. 2013; NICE 2014; Grunze et al. 2013; Goodwin et al. 2016). Monotherapy and adjunctive therapy with aripiprazole and risperidone long-acting injection also appear to be effective maintenance treatments, but primarily for the prevention of recurrent manic episodes, while lamotrigine is recommended primarily for prevention of recurrent depressive episodes.

Among patients in the community receiving long-term treatment for bipolar disorder, approximately 50% have been reported to experience a recurrence within 2 years (Tohen et al. 2003) even when receiving guideline-based treatment from clinicians with specialized training in the pharmacological management of bipolar disorder (Perlis et al. 2006). Thus, there is clearly a need for additional treatment options that are safe, well tolerated, and effective for the maintenance therapy of bipolar disorder.

Lurasidone, in the dosing range of 20–120 mg/day, has been approved in the USA and Canada for the treatment of acute bipolar depression, both as monotherapy and as adjunctive therapy with lithium or valproate, based on the results of 2 positive randomized, double-blind, 6-week trials (Loebel et al. 2014a, 2014b). In a 6-month, open-label extension study (Ketter et al. 2016), treatment with lurasidone was found to be well tolerated and had minimal effects on weight, lipids, and measures of glycemic control. In this open-label extension trial, acute improvement in depressive symptoms was maintained during 6 months of additional lurasidone therapy, with more than 90% of patients who met responder criteria at baseline of the extension study continuing to meet responder criteria at month 6, and the majority of initial non-responders achieving responder status by 6 months in both the monotherapy group (83%) and in the adjunctive therapy group (73%). Furthermore, treatment with lurasidone, without use of adjunctive mood stabilizers, was associated with a low rate of mania (1.3%) during the 6-month treatment period.

We now report the results of a continuation study in which patients who completed the 6-month extension study summarized above (Ketter et al. 2016) received up to 18 months of additional open-label treatment to evaluate the safety, tolerability, and effectiveness of long-term treatment with lurasidone.

## Methods

This was an 18-month, open-label, continuation study (clinicaltrials.gov identifier: NCT01485640) designed to monitor the long-term safety, tolerability, and effectiveness of lurasidone in patients who initially met DSM-IV-TR criteria for either chronic schizophrenia or bipolar I depression. The current analysis is limited to the group of patients who participated in this study with a bipolar disorder diagnosis; patients with a diagnosis of chronic schizophrenia are not included in the current report and will be reported separately.

Patients with bipolar depression were initially enrolled in one of three 6-week, double-blind, placebo-controlled trials [monotherapy with lurasidone, 1 study; clinicaltrials.gov identifier: NCT00868699 (Loebel et al. 2014b); adjunctive therapy with lurasidone and lithium or valproate, 2 studies; clinicaltrials.gov identifiers: NCT00868452 and NCT01284517 (Loebel et al. 2014a; Suppes et al. 2016)], followed by a 6-month open-label extension study of lurasidone in flexible daily doses of 20–120 mg [clinicaltrials.gov identifier: NCT00868959; Ketter et al. 2016]). Six-month study completers were then treated for an additional 18 months with flexible, once-daily doses of lurasidone in the range of 20–80 mg. Concomitant therapy with mood stabilizers and antidepressant medications was permitted throughout the open-label studies.

Eligible patients could enroll into this continuation study directly after completing the 6-month extension study. Patients who had completed the extension study prior to the initiation of the current protocol at a study site were also permitted to enroll for up to 3 months after completion of the prior extension study (*N* = 34; 27.9% of the patients analyzed in the current report).

Patients were included in the current extension study if they were judged by the Investigator to be suitable for participation in an 18-month, open-label study, able to comply with the protocol, and not at imminent risk of suicide, or injury to self or others. Patients were excluded who answered “yes” to “suicidal ideation” items 4 or 5 on the Columbia Suicide Severity Rating Scale (C-SSRS).

The study was conducted from March 2012 to February 2014 at 48 centers in 12 countries in North and South America (*N* = 9), Europe (*N* = 67), Asia (*N* = 32), and Africa (*N* = 14). The study protocol was approved by Independent Ethics Committees associated with each study center. Prior to entering the current 18-month continuation study, an informed consent form was reviewed and signed by all patients. Study conduct was in accordance with Good Clinical Practices as required by the International Conference on Harmonization guidelines and in accordance with ethical principles of the declaration of Helsinki of 1975 (as revised in 1983). The 18-month study was discontinued for administrative reasons on 7 February 2014 by the Sponsor after lurasidone became available in the regions in which the study was conducted.

Patients who met entry criteria were treated with flexible doses of lurasidone in the range of 20–80 mg/day. The dose of lurasidone could be adjusted at any time, based on the clinical judgment of the Investigator, to optimize efficacy and tolerability. Patients were instructed to continue the dosing regimen from the previous lurasidone extension study (in the evening or morning within 30 min of eating). At each visit, patients were dispensed up to a 3-month supply of study medication, based on investigator judgment.

Patients were permitted to be treated concomitantly with benzodiazepines, mood stabilizers (e.g., lithium, divalproex, or lamotrigine), or antidepressants at the discretion of the Investigator. Monoamine oxidase inhibitors and antipsychotic medications (other than lurasidone) were prohibited.

### Safety and effectiveness evaluations

Safety and effectiveness evaluations were performed at 3-month intervals, with additional visits scheduled if indicated due to adverse events or clinical worsening. The incidence and severity of treatment-emergent adverse events were recorded. Movement disorders were assessed using the Barnes Akathisia Rating Scale (BARS; Barnes 1989), the Simpson-Angus Scale (SAS; Simpson and Angus 1970), and the Abnormal Involuntary Movement Scale (AIMS: Guy 1976). Additional safety evaluations included weight, vital signs, hematology, chemistries, and urinalysis. Suicidal ideation and behavior were assessed using the Columbia Suicide Severity Rating Scale (C-SSRS; Posner et al. 2011). The effectiveness of lurasidone was assessed using the Clinical Global Impressions Bipolar Version, Severity of illness scale (CGI-BP-S; Spearing et al. 1997) at Baseline of the acute double-blind and 6-month extension studies; and the Clinical Global Impression, Severity scale was utilized in the current 18-month continuation study (CGI-S; Guy 1976). Both scales provide an overall rating of illness severity on a 7-point severity scale, with the CGI-BP-S providing bipolar-specific prompts.

### Statistical analyses

The safety population consisted of all patients who received at least one dose of lurasidone in the 18-month continuation study that followed the initial 6-week double-blind study and the subsequent 6-month open-label extension study. The primary safety analyses consisted of the incidence of treatment-emergent adverse events, serious adverse events, and discontinuations due to adverse events. Descriptive statistics were also calculated for the following safety variables: body weight, proportion of patients with ≥7% weight change from baseline, body mass index (BMI), vital signs, movement disorders as assessed by the BARS, SAS, and AIMS scales, as well as laboratory results (chemistries, hematology, and urinalysis). Mean (SD) change in the CGI-BP-S and CGI-S scores were reported from the baseline of the original double-blind acute treatment study based on both an observed case (OC) and last observation carried forward to endpoint (LOCF-endpoint) analysis. Treatment response was defined as achieving a CGI-S score of ≤2 (mild severity to no symptoms); remission was defined as a CGI-S score = 1 (no symptoms). A post hoc Kaplan–Meier survival analysis was performed to estimate probability of relapse during 18 months of open-label treatment with lurasidone. Relapse was defined as meeting one of the following three criteria: (1) a 1-point increase over continuation baseline in the CGI-severity score; (2) reported adverse event of treatment-emergent hypomania, mania, or depression; or (3) discontinuation due to treatment-emergent clinical worsening or insufficient clinical response.

## Results

**Fig. 1:**
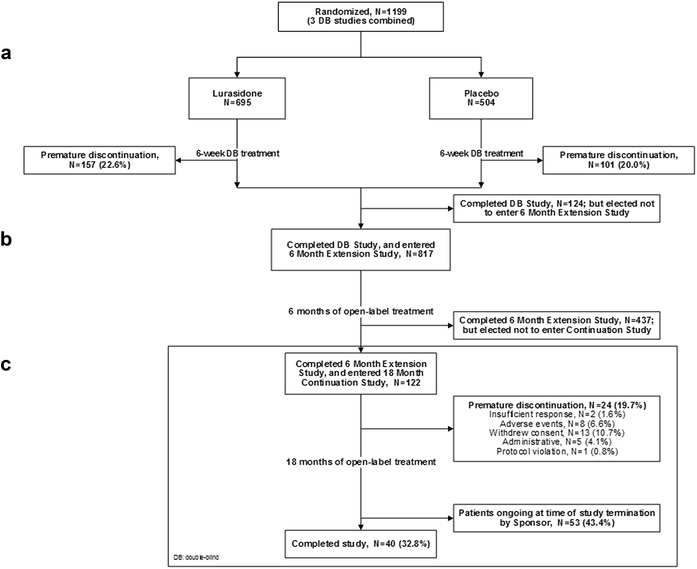
Patient disposition. **a** Acute phase (6 weeks).** b** Extension phase (6 months).** c** Continuation phase (18 months)

In the three 6-week, double-blind, placebo-controlled acute bipolar depression treatment studies combined, a total of 941/1199 patients (78.5%) were completers (lurasidone, 77.4%; placebo, 80.0%), of whom 817/941 (86.8%) entered the 6-month extension study, and 559/817 (68.4%) completed the extension study. A total of 122/559 patients (21.8%) provided informed consent and entered the current 18-month continuation study, of whom 40/122 (32.8%) were completers (Fig. 1). Fifty-eight of the 82 patients (70.7%) who discontinued from the study (out of a total sample of 122) were patients who were ongoing at the time the study was terminated by the sponsor; all of these patients had received at least 6 months of treatment prior to study termination. The remaining 24 patients discontinued due to adverse event (*N* = 8, 6.6%), insufficient clinical response (*N* = 2; 1.6%), withdrawal of consent (*N* = 13; 10.7%), and protocol violation (*N* = 1; 0.8%).

Baseline demographic and clinical characteristics for patients in the current continuation study are summarized in Table 1. Baseline characteristics in the 3 initial double-blind, 6 week studies, and the current 18-month continuation study were as follows: proportion of males (46.7 and 52.5%, respectively), whites (64.0 and 58.2%, respectively), use of adjunctive mood stabilizers (58.2 and 76.2%, respectively), mean age (42.2 and 41.3 years, respectively), and age of diagnosis with bipolar disorder (28.5 and 31.3 years old, respectively).

**Table 1:** Baseline demographic and clinical characteristics of patient sample at 18-month continuation study baseline (safety population)

Characteristic	Lurasidone (*N* = 122)	
	*N*	%
Male	64	52.5
*Race*		
White	71	58.2
Black/African–American	3	2.5
Asian	32	26.2
Other	16	13.1
History of rapid cycling (≥ 4 episodes in past 12 months)	7	5.7
*Adjunctive mood stabilizer, n (%)* ^a^		
Yes	93	76.2
No	29	23.8

Characteristic	Lurasidone (*N* = 122)	
	Mean	SD
Age, years	41.3	12.2
Age at initial bipolar diagnosis, years	31.3	11.8
Duration of depressive episode (at entry into DB study), weeks	10.8	7.2
*MADRS total score*		
Acute double-blind baseline	28.8	4.4
6-month extension baseline	13.8	8.7
18-month continuation study baseline	6.5	5.3
*CGI*-*severity score* ^b^		
Acute double-blind baseline	4.3	0.5
6-month extension baseline	2.8	1.0
18-month continuation study baseline	2.1	1.0

^a^ In acute double-blind (DB) study

^b^ Note that the CGI-BP-S scale was used to assess severity at Baseline of the acute DB and 6-month extension studies

Among the 122 patients treated with lurasidone in the current study, 93 (76.2%) were receiving adjunctive therapy with one of several formulations of valproic acid (*n* = 58) or lithium (*n* = 35). Additional concomitant medications included acetaminophen (12.3%), lorazepam (9.0%), zolpidem (6.6%), levothyroxine (4.9%), trihexyphenidyl (4.1%), and venlafaxine (4.9%).

The mean (SD) daily dose of lurasidone during the current 18-month study was 61.8 (17.5) mg and was similar in the monotherapy and adjunctive therapy groups. The modal daily dose of lurasidone for the combined therapy groups was 20 mg for 9.0% of patients, 40 mg for 11.5% of patients, 60 mg for 43.4% of patients, and 80 mg for 36.1% of patients Table 2.

**Table 2:** Change in weight and BMI from double-blind acute phase baseline

	Baseline *N* = 122			Mean change (from double-blind acute phase baseline)			
	6-week double-blind mean	6-month extension mean	Current study mean	Month 12 (OC)		Month 24 (OC)	
				*n*	Mean	*n*	Mean
Weight, kg	75.4	75.6	76.3	113	+1.8	55	+0.8
BMI, kg/m^2^	26.6	26.7	27.0	113	+0.7	55	+0.3

Month 12 = 6 months of extension +6 months of maintenance treatment; month 24 = 6 months of extension +18 months of continuation treatment (patients randomized to lurasidone in the initial double-blind acute treatment study had an additional 6 weeks of exposure to lurasidone)

*BMI* body mass index, *OC* observed case analysis

### Safety

During the 18-month continuation phase, at least one treatment-emergent adverse event was reported by 42.6% of patients, with 4.9% of patients rating at least one event as “severe.” The most frequent adverse events were headache (7.4%); diarrhea, influenza, and nasopharyngitis (4.9% each); depression and vomiting (4.1% each); increase in hepatic enzymes, mania, nausea, and viral upper respiratory infection (3.3% each); and parkinsonian symptoms (2.5%). One patient each (0.8%) reported sedation and somnolence. Five patients (4.1%) reported extrapyramidal symptoms (Parkinsonism and tremor; no patients reported akathisia).

In addition to the 3 patients noted above who discontinued due to treatment-emergent mania or suicidal ideation, 5 patients also discontinued due to treatment-emergent adverse events: 1 patient each due to mania and hypomania, 1 patient each due to diabetes and hyperglycemia, and 1 patient due to an increase in hepatic enzymes.

Six patients experienced a serious adverse event during the course of study treatment: one patient with a pilonidal cyst, one patient with a fracture of the first and second metatarsals, two patients with treatment-emergent symptoms of mania (both were discontinued from the study), and two patients with treatment-emergent depressive symptoms, one of whom reported suicidal ideation and was discontinued from the study. There were no deaths in the study. No active suicidal ideation was identified on the C-SSRS during the course of the study.

Eighteen months of treatment with lurasidone was associated with a mean change, from double-blind baseline to LOCF-endpoint, of −0.1 on the BARS global score, +0.02 on the SAS 10-item mean score, and −0.1 on the AIMS total score.

The mean weight, at baseline of the 6-week double-blind trial, in the subgroup of patients (*N* = 122) who completed the subsequent 6-month extension study and entered the 18-month continuation study, was 75.4 kg, and the mean BMI was 26.6 kg/m^2^. Among patients who completed 24 months of overall treatment (*N* = 55), mean change in weight and BMI was +0.8 kg and +0.3 kg/m^2^, respectively. Among patients who completed 12 months of treatment in the continuation study (*N* = 118;), mean change in weight and BMI was +1.8 kg and +0.7 kg/m^2^, respectively, with 16.1% having a weight gain of ≥7, and 5.9% having a weight loss of ≥7%.

Small median changes from double-blind baseline were also observed at months 12 and 24 in metabolic parameters and prolactin (Table 3). No clinically significant changes were observed for heart rate, orthostatic blood pressure (systolic or diastolic), or respiratory rate.

**Table 3:** Change in laboratory parameters from double-blind acute phase baseline

	Baseline means			Median change (from double-blind acute phase baseline)			
	6-week double-blind *N* = 122	6-month extension *N* = 120–122	Current study *N* = 98–103	Month 12 (OC)		Month 24 (OC)	
				*n*	Median	*n*	Median
Total cholesterol, mg/dL	195.5	188.7	197.9	111	+5.0	54	−3.0
LDL cholesterol, mg/dL	116.8	110.2	117.5	108	−4.5	53	−10.0
HDL cholesterol, mg/dL	50.5	50.4	50.3	112	1.0	54	−1.0
Triglycerides, mg/dL	151.5	151.0	157.6	111	+10.0	54	+26.0
Glucose, mg/dL	91.1	93.1	93.7	112	+2.5	54	+3.5
HbA1c, %	5.3	5.3	5.4	112	0.0	53	−0.1
Prolactin, ng/mL	10.9	13.1	13.7	112	+0.8	54	+0.6
Prolactin, males	10.1 (*N* = 64)	9.5 (*N* = 64)	8.3 (*N* = 52)	59	−0.2	27	+0.2
Prolactin, females	11.7 (*N* = 58)	17.3 (*N* = 57)	19.3 (*N* = 51)	53	+1.4	27	+0.6

Month 12 = 6 months of Extension + 6 months of Maintenance treatment; Month 24 = 6 months of Extension + 18 months of Continuation treatment (patients randomized to lurasidone in the initial double-blind acute treatment study had an additional 6 weeks of exposure to lurasidone)

*HbA1c* glycosylated hemoglobin, *LDL* low-density lipoprotein, *HDL* high-density lipoprotein, *OC* observed case analysis

### Effectiveness

Improvement in bipolar illness severity observed on lurasidone during initial acute and extension phase therapy was maintained in the great majority of patients across 18 months of continuation treatment. At initial double-blind Baseline, the mean CGI-BP-S score was 4.3. After 6 weeks of double-blind treatment, the mean CGI-BP-S score was 2.7 in the lurasidone group and 2.9 in the placebo group. After 6 months of extension treatment (Baseline of the current continuation study), the mean CGI-BP-S score was 2.1. This level of improvement was maintained through 18 months of continuation treatment (a minimum of 24 months of total treatment), with a mean CGI-Severity score of 2.0 at month 12 (OC), 1.8 at month 18 (OC), 1.7 at month 24 (OC), and 1.9 at LOCF-endpoint.

**Fig. 2:**
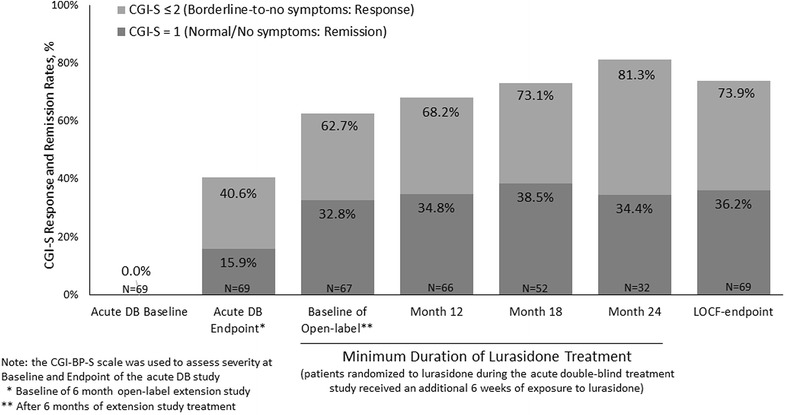
Response and remission rates during 24 months of treatment based on cgi-severity criteria

Across 18 months of continuation treatment, global depression symptom severity remained at a low level of severity, with 63–81% of patients reporting clinical response, defined as borderline-to-no symptoms (CGI-S ≤ 2), and 33–39% reporting full symptom remission (CGI-S = 1; Fig. 2).

In a post hoc Kaplan–Meier analysis, the estimated probability of relapse on lurasidone monotherapy and adjunctive therapy at 6 months was 14.6 and 15.4%, respectively; at 12 months, it was 18.3 and 23.4%, respectively; and at 18 months, it was 18.3 and 29.1%, respectively.

## Discussion

This was an 18-month, open-label continuation study designed to evaluate the long-term safety and tolerability of lurasidone (20–80 mg/day) in patients with bipolar depression who had completed 6 weeks of treatment with lurasidone or placebo in a randomized, double-blind trial, followed by 6 months of open-label extension phase treatment on flexible doses of lurasidone in the dosing range of 20–120 mg/day. Concomitant therapy with mood stabilizers was permitted in both open-label studies and in 2 of the 3 acute double-blind studies. Effectiveness was also evaluated as a secondary outcome with a focus on maintenance of effect.

The results of the current study suggest that up to 2 years of treatment with lurasidone, in the daily dose range of 20–80 mg, is associated with a relatively low potential for weight gain and adverse metabolic effects. Changes in weight, BMI, lipids, glycemic indices, and prolactin were small, and not clinically meaningful. These long-term data confirm and extend the findings obtained in previous long-term trials in schizophrenia and bipolar disorder indicating that lurasidone has a low propensity for adverse effects on weight, lipids, and glucose metabolism (Citrome et al. 2012; Loebel et al. 2013; Meyer et al. 2015; Tandon et al. 2016; Ketter et al. 2016; Correll et al.^2016^). It is well-established that bipolar disorder is associated with approximately a twofold increased risk of cardiovascular mortality (Osby et al. 2001; Crump et al. 2013; Miller and Bauer 2014), attributable to various factors, including significant increased risk of obesity, hyperlipidemia, diabetes, and metabolic syndrome (Goldstein et al. 2011; McElroy and Keck 2014). Evidence suggests that treatment with many widely used atypical antipsychotics is associated with adverse cardiometabolic effects that increase mortality risk (Correll et al. 2015). Lurasidone would appear to provide an important long-term treatment option, given its low propensity for adverse effects on weight and metabolic parameters.

Discontinuation due to adverse events during 18 months of treatment with lurasidone was relatively low (6.6%). Adverse event rates were notably lower than rates previously reported for lurasidone during acute treatment (Loebel et al. 2014a, b), with only one adverse event (headache, 7.4%) occurring with an incidence above 5%.

Extrapyramidal symptom-related adverse events were reported in a smaller proportion of patients in the current study (4.1%) than in previously reported long-term treatment studies in schizophrenia (Correll et al. 2016). Consistent with this, use of anticholinergic medication was relatively low (4.1%), and minimal effects were noted on movement disorder measures.

Overall, the adverse event profile of lurasidone in this bipolar population was comparable to what has been reported in previous long-term studies of lurasidone in patients with schizophrenia (Correll et al. 2016).

Improvement in depressive symptoms was maintained on lurasidone in the great majority of patients. At baseline of the current study, the mean CGI-Severity score was 2.1. The mean CGI-S score remained below 2 (“borderline mentally ill”) on both OC and LOCF-endpoint analyses. The proportion of patients reporting remission of manic or depressive symptoms during 18 months of treatment was in the range of 35–38%, with remission defined based on stringent CGI-Severity criteria (CGI-S = 1) representing the absence of bipolar symptomatology.

The results of a Kaplan–Meier analysis found a low estimated probability of relapse during 18 months of continuation treatment with lurasidone, both as monotherapy (18.3%) and adjunctive with lithium or valproate (29.1%). These results compare favorably to naturalistic studies, with 1.5- to 2-year follow-up, of bipolar patients receiving standard maintenance therapy in the community, which typically report rates of recurrence of depression or mania that are in the range of 40–60% (Judd et al. 2002; Tohen et al. 2003; Joffe et al. 2004; Perlis et al. 2006). Double-blind, placebo-controlled recurrence prevention trials in bipolar patients also report recurrence rates during 1.5–2 years of treatment with atypical antipsychotics that were somewhat higher than the current findings, in the range of 18–31% (Tohen et al. 2004; Vieta et al. 2008; Suppes et al. 2009).

### Study limitations

Study limitations included the open-label study design, lack of concurrent placebo or other control group, the small sample size, and the relatively small proportion of patients who completed the study. A total of 71% of patients who discontinued prematurely were ongoing in the study for at least 6 months, but were discontinued due to study termination by the sponsor. Other potential limitations include the use of an enriched sample of patients who had demonstrated tolerability and responsivity to lurasidone during up to 7.5 months of initial lurasidone therapy, and reliance on relatively minimal efficacy measures.

In summary, up to 2 years of treatment with lurasidone (monotherapy or adjunctive therapy) was found to be safe and well tolerated in this bipolar I disorder population that presented initially with a major depressive episode. Consistent with previous long-term trials in patients with a diagnosis of schizophrenia, minimal effects on weight and metabolic parameters were observed. Improvement in depressive symptoms was maintained in the majority of patients during long-term lurasidone treatment, with relatively low rates of relapse.

## Abbreviations

AIMS: Abnormal Involuntary Movement Scale
BARS: Barnes Akathisia Rating Scale
BMI: body mass index
CGI-BP-S: Clinical Global Impressions Bipolar Version, Severity of illness scale
CGI-S: Clinical Global Impression, Severity scale
C-SSRS: Columbia Suicide Severity Rating Scale
HbA1c: glycosylated hemoglobin
LOCF: last observation carried forward
OC: Observed case
SAS: Simpson-Angus Scale
SD: standard deviation
